# Peer Support Supervision Competencies: Results of Participatory Action Research

**DOI:** 10.1007/s10597-026-01612-x

**Published:** 2026-04-16

**Authors:** Jonathan P. Edwards, Amy B. Spagnolo, Rita Cronise, Gita Enders, Joanne Forbes, Carlos Pratt

**Affiliations:** 1 School of Social Work, Columbia University, New York, USA; 2 Department of Psychiatric Rehabilitation and Counseling and ScarletWell, Rutgers, The State University of New Jersey, New Brunswick, USA; 3 Office of Behavioral Health, New York City Health and Hospitals Corporation, New York, USA

**Keywords:** Peer support specialist, Supervision, Competencies, Training, Model

## Abstract

Peer Support Specialists (PSS) are a rapidly expanding workforce in behavioral healthcare, with over 100,000 practitioners currently active in the U.S. Despite the evidence-base for peer services, supervision remains a significant challenge, often leading to role confusion when managed by non-peer supervisors. This study sought to identify specific supervision competencies that account for unique considerations of PSS. This study utilized a multi-phase Participatory Action Research (PAR) approach, employing online Delphi focus groups and a nationwide pilot survey to identify and validate specific competencies for PSS supervision. Assessing the importance, criticality, and frequency of 71 supervisory tasks across five functions of supervision (Administrate, Support, Educate, Advocate, and Evaluate), identified by 100 PSS and supervisors, yielded the 25 supervisory competencies rated highest and lowest, aligned with the Five Critical Functions of Supervision model. Survey results revealed a high prioritization for tasks that support role clarity, active listening, and leading through empathy. These findings provide an empirical schema for developing standardized training curricula and practice guidelines for ensuring effective and efficient PSS supervision. Results suggest that further research on the competencies of the Five Critical Functions of Supervision model is warranted.

Peer Support Specialists (PSS) are individuals who have been successful in the recovery process from mental health conditions and use their lived experience to assist others in similar situations. Through shared understanding, respect, and mutual empowerment, these workers help individuals become and remain engaged in the recovery process (University of Illinois Chicago, Center on Mental Health Services Research and Policy, 2025; SAMHSA, 2017). PSS comprise a rapidly growing workforce within behavioral healthcare (Wolf, 2018). The number of working PSS has trended upward for decades with recent estimates suggesting more than 100,000 PSS are active in the U.S. (University of Illinois Chicago, 2025). In 2020, SAMHSA (2020) noted exponential growth of the peer support workforce and projected a need for over 1.1 million PSS and 308,622 PSS supervisors, thereby solidifying peer support as a recognized component of the mental health landscape. PSS work in diverse settings ranging from independent peer-run agencies and care management teams to traditional clinical environments, crisis services, inpatient services, and psychosocial clubhouses (Cronise et al., 2016; Myrick & del Vecchio, 2016; Salzer et al., 2010).

In 2007, the Centers for Medicare and Medicaid Services (CMS) (2007) recognized peer support as an evidence-based service, making it reimbursable under state Medicaid plans. This recognition requires state-specific training and certification, and as of 2023, every state except South Dakota had established such programs (Peer Recovery Center of Excellence, 2023). Furthermore, CMS criteria mandated that PSS be supervised by a competent, qualified mental health professional as defined by each state with a 2024 update to include “approved experienced peers” (Smith, 2007; CMS, 2024). Despite the evidence-base and demonstrated effectiveness of peer support services (Chinman et al., 2014a; Mental Health America, 2018), the supervision of PSS is fraught with significant challenges (Forbes et al., 2022; Stefancic et al., 2021). Many supervisors lack personal lived experience, are primarily clinically oriented, and remain unfamiliar with the core values of peer support. This frequently leads to role confusion, a lack of understanding regarding the power of personal stories, and insufficient time dedicated specifically to supervision (SAMHSA, n.d.). Given the importance of supervision as a key to successful integration, job satisfaction, and retention of peer support workers (Reuling et al., 2024; SAMHSA, 2023; Abraham et al., 2021; Cronise et al., 2016; Delman & Klodnick, 2016; Davis, 2015; & Davis, 2013) this study utilized an investigative frame, The Five Critical Functions of Supervision (herein also referred to as the “five-function model”) to articulate the specific supervision competencies associated with these functions, whereby supervisory tasks are categorized under five functions: administrative, supportive, educative, advocative, and evaluative (Edwards, 2025; Edwards et al. 2024; Edwards, 2024; Edwards & Foglesong, 2024; Edwards, 2023; Edwards et al., 2022; Edwards & Ikuta, 2016). The aims of this research were to (1) identify perspectives on supervision from both PSS and supervisors; (2) explore the breadth and depth of five supervision functions by identifying competencies associated with these functions; and (3) determine the importance, criticality, and frequency of supervision competencies identified by subject matter experts.

## Review of the Literature

While research on traditional clinical supervision is robust, supervision of peer support specialists remains a developing area of exploration. A recent study by Forbes et al. (2022) discussed the challenges that occur when peer support workers are supervised by non-peer supervisors. This leads to role confusion (Alberta et al., 2012; Cabral et al., 2014; Hino, 2014), lack of understanding of the core values of peer support (Fogelsong et al. 2022a; N.A.P.S., 2019; White et al., 2020), little or no time dedicated to supervision (Foglesong et al., 2022a, b), and the lack of understanding of the power of personal stories (Mancini, 2019). The systemic issues affecting the PSS workforce include lack of opportunities for professional advancement within peer support services, which oftentimes necessitates seeking advancement opportunities in other disciplines (Bennetts et al., 2013). Low salaries and lack of pay equity are often expressed concerns of PSS (Cronise et al., 2016; Miyamoto & Sono, 2012; Mowbray et al., 1996). Peer drift, the erosion of peer identity through cooptation into clinical cultures (Edwards & Solomon, 2023; Chinman et al., 2014b; Moran et al., 2013), occurs when PSS roles and tasks are not understood and supported by supervisors.

Literature identifying the specific competencies associated with the supervision of peer support workers includes the Pillars of Peer Supervision (Daniels et al., 2015), and the National Practice Guidelines for Peer Support Specialists and Supervisors (NPG-S) (Foglesong et al. 2022a, b; N.A.P.S., 2019). Despite these resources, specific supervisory functions have not been defined to ensure optimal support of PSS and quality peer delivered services given the important differences between peer-delivered services and traditional clinical practice (Forbes et al., 2022; Mead & McNeil, 2004; Mead et al., 2001; N.A.P.S., 2013, 2019; SAMHSA, 2015). The available literature does include suggestions for PSS supervision and outcomes associated with effective supervision. SAMHSA’s Supervision of Peer Workers: Bringing Recovery Supports to Scale Technical Assistance Center Strategy (BRSS TACS) report promotes a three-function model of supervision based on the 1992 textbook by Alfred Kadushin, *Supervision in Social Work* (SAMHSA BRSS TACS, 2018). The three core functions of supervision identified were administrative, educative, and supportive. Based on the available research on supervision of PSS and feedback from supervisors and peer support workers, Edwards expanded the three-function model to include advocative and evaluative resulting in the Five Critical Functions of Supervision, which were validated through this research (Edwards, 2025; Edwards et al., 2024; Edwards, 2024; Edwards & Foglesong, 2024; Edwards, 2023; Edwards et al., 2022; Edwards & Ikuta, 2016). The advocative function describes the supervisor’s task in advancing knowledge and safeguarding the integrity of the peer support worker role and promoting the value of the peer support discipline. The evaluative function describes the supervisor’s role in articulating clear expectations regarding the work and developing methods for assessing performance. Using this framework, the supervisor’s charge is to administrate by focusing on role clarity and organizational orientation; to educate by modeling peer values and discussing the history of the discipline; to support the PSS by building trust through mutuality and active listening; to advocate by advancing knowledge within the organization of the PSS role and addressing power differentials; and to evaluate by articulating clear expectations and utilizing collaborative assessment methods.

### Methods

**Fig. 1:**
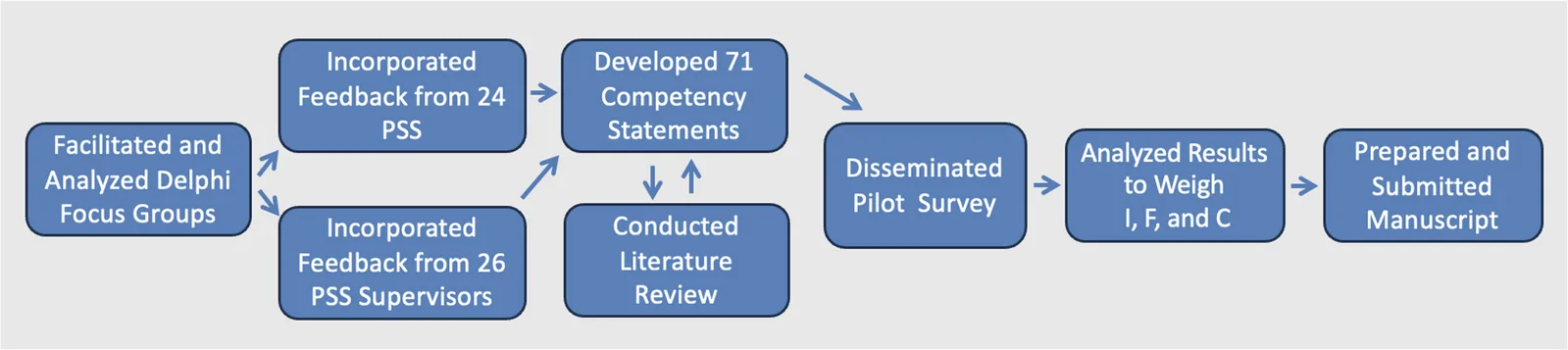
Research Method

This study employed the core principles of Participatory Action Research (PAR) through the active and sustained involvement of PSS and supervisors as co-constructors of knowledge, rather than as passive research subjects (see Fig. 1). Across multiple phases of data generation, analysis, and refinement of competency statements, PSS and supervisors were instrumental in each iterative process. The iterative, consensus-building process, participant review of draft competency statements, and successive rounds of revision, demonstrates a standard PAR procedure. The findings are explicitly intended to inform training, the practice of peer support supervision, and workforce development. By eliciting subject matter expertise and engaging participants in shaping and assessing both the content and application of supervision competencies, the study aligns with PAR’s practice-focused aims, ensuring that outcomes are directly relevant and responsive to the community under study.

The identification and evaluation of peer support supervisor competencies was a multi-phase procedure that included (1) facilitation of focus groups to identify panelists’ views on the competencies required for PSS supervision structured by the Five Critical Functions of Supervision model, (2) response analysis and iterative consensus building among supervisors and PSS on identified competencies, and (3) the development, dissemination and analysis of an online nation-wide pilot survey by a PAR e-Delphi group. Participating supervisors and PSS evaluated the importance, frequency, and criticality of each identified competency.

### e-Delphi Focus Groups

An e-Delphi method for focus group facilitation was employed in this study. The e-Delphi technique uses a combination of qualitative and quantitative processes that draw on the opinions of selected subject matter experts (SMEs) and aims to obtain group consensus on a topic via an online software platform (ie.Zoom) (Hasson et al., 2000; Iqbal & Pipon Young, 2009; Janke et al., 2016). e-Delphi methods are appropriate when scientific knowledge on the topic under investigation is scarce (Crutzen et al., 2008; Iqbal & Pipon Young, 2009), and the electronic survey method for collecting expert opinions is useful when face-to-face data collection is impractical (Green & Kreuter, 1999). The e-Delphi process allows for obtaining expert opinions without physically bringing experts together. The e-Delphi technique has been effectively used to examine core competencies in health promotion (Battel-Kirk et al., 2009), crisis peer support competency development (Karyczak et al., 2025), and is well suited to investigating health issues (Hasson et al., 2000). This method was chosen because while there is growing interest and a definitive need for quality peer support supervision, there is little consensus regarding the core competencies.

### Participants

Focus group panelists were identified by the research team based on their experiential, regional, and demographic diversity and invited to participate in a training needs assessment for supervisors of PSS. Panelists were largely identified based on their participation in a Supervision Community of Practice (CoP) and/or their collaboration with the research team. Inclusion criteria required that the panelist was currently supervising a peer support specialist. Once panelists agreed to participate, they were assigned to a focus group based on their demographics and geographic region and provided a link to join the virtual session. In total, 26 panelists participated in the four focus groups (Grp 1 = 5, Grp 2 = 6, Grp 3 = 5, Grp 4 = 10; total *n* = 26). This purposive sampling of panelists allowed the research team to gather information-rich “cases” and make the most out of the focus group method (Nyumba et al., 2018). The 24 participants who shared their demographic information were 42% male, 54% female and 4% non-binary. They were 50% White or Caucasian, 21%on Brown, Black, or African American, 8% Hispanic, Latinx, or Spanish origin, and 21% listed themselves as other. Ages ranged from 25 to 64 with the modal age in the 45 to 54 range. 67% (*n* = 16) of the participants had over five years of supervisory experience, 29% (*n* = 7) had between three and five years of experience, and one participant had between one and two years of supervisory experience. 78% (*n* = 18) reported lived experience of mental illness. 58% (*n* = 14) reported holding peer support specialist certification. All reported at least some college with 13% (*n* = 3) holding an associate’s degree, 17% (*n* = 4) holding a bachelor’s level degree, 58% (*n* = 14) a master’s degree, and one participant with a doctoral degree.

#### Instruments: PSS Supervision Focus Group Protocol and Script; Focus Group Organizer; and Supervision Competency Statement Chart

The focus groups were virtually led by two researchers and one other member of the team who served as a note taker. All sessions occurred via zoom with the audio/video recording and text transcription features utilized. e-Delphi focus group materials included a research protocol for focus group facilitation and an accompanying script utilized by the researchers for each of the four virtual focus groups. The protocol included a welcome and introduction to the 90-minute group and described the role of the panelists. The five-function supervision model was described and its use as a method to organize information on the supervision of peer support specialists clarified. Panelists were told that their responses would inform the development of a survey instrument to identify and assess the importance, frequency, and criticality of supervisory functions, with the goal of improving peer support supervision and creating a curriculum for online supervision training.

The researchers also utilized a focus group response organizer (NMAC, 2015; Slocum, 2005) to capture feedback from panelists and to compare statements and impressions in the thematic analysis. This form included the question, spaces for each panelist’s response, sections for any follow up statements, a column to capture any themes the researchers heard in the responses, and a column for any observations made by the researchers when the panelists spoke. Like the *Focus Group Response Organizer*, the research team also utilized a *Supervision Competency Statement Chart* to capture the statements that the team arrived at via consensus. The *Supervision Competency Statement Chart* included one section for each of the five functions of supervision, one column for the draft competency statement, a second column entitled “Suggested Revisions” with the instruction to “Use this column to suggest a revision to the language of the competency” and a third column entitled “Suggested Additions” with the instruction to “Use this column to add any additional competency not included in the original list”.

In each focus group, the term competency was defined as a “critical work function or task”. This definition was repeated throughout the focus groups to re-orient the panelists to the topic. Panelists were asked a total of six questions. The opening question, “In just a few words, what is the biggest reason you agreed to join us today to talk about peer specialist supervision?” was used as an engagement question. One question for each supervision function was then asked, “When you think of the term (administrative/evaluative/educative/supportive/advocative) supervision, what competencies come to mind?”. The panelists were then provided a summary and informed they would receive the draft competency statements for further revision and additions via online link.

Because the protocol was designed for the purposes of curricula development and needs assessment, consent to participate in this discussion was not required. IRB approval to analyze archival data was secured for this publication. All information collected was de-identified and the data is presented in aggregate form. Panelists’ responses were coded, and no information was shared with any party other than the focus group facilitators and other members of the research team. The virtual focus groups were also recorded, and the Zoom transcriptions were saved for verbatim coding and analyses by all members of the research team.

#### Procedure: Focus Group Analysis & Consensus Building

Focus group data were analyzed using inductive constant comparison to generate and refine supervision competencies directly from participant input. Research team members reviewed focus group transcripts, written notes, and post-session participant feedback in an iterative, consensus-building process, comparing data within and across groups to identify recurring ideas, assess saturation, and develop competency statements (Strauss & Corbin, 1998; Onwuegbuzie et al., 2009). As analysis progressed, previously generated statements were continually revisited, revised, merged, or clarified in response to new data. Only after competencies had been inductively derived were they organized using the five-function model, which served as a post-analytic organizing framework rather than a deductive coding structure.

What differentiates this approach from classic grounded theory is that the five-function model was not used to generate categories during initial coding, but rather to organize inductively derived competencies at a later analytic stage. Thus, constant comparison was used to develop and refine the content of competencies, while the five-function model functioned as a post-hoc organizing structure to support interpretation, consensus building, and applied use in survey development. After the four focus groups were facilitated, the team determined that no new competencies had been identified and that the list of competencies was reflective of all panelists’ input.

Research team consensus was achieved through a systematic, iterative process. Following each focus group, members of the research team independently reviewed transcripts, written notes, and response organizers to identify supervision competency statements. The team then met to compare interpretations, discuss similarities and differences, and refine the wording and scope of emerging competencies. Disagreements were addressed by returning to the original data, examining participants’ language and examples, and deliberating until shared agreement was reached regarding the meaning and representation of the data. As additional focus groups were analyzed, previously developed competency statements were revisited and revised as needed, allowing the team to assess convergence and saturation across groups. This process continued until the research team determined that no substantively new competencies were emerging and that the final set adequately represented the collective focus group data.

During the subsequent phase, competency statements were grouped into the five supervisory functions and reviewed by the research team to establish conceptual alignment and consistency in wording. Following completion of the Delphi consensus rounds, the refined set of statements was shared with the original focus group participants and a group of peer support specialists (PSS), recruited through snowball sampling, to solicit broader feedback on clarity, relevance, and completeness. Feedback from this phase informed minor wording refinements but did not alter the core competencies that had achieved consensus through the Delphi process. A total of 24 people were involved in this phase of competency statement construction. Following each iteration, the responses were analyzed, and the statements were revised accordingly. This iterative method generated feedback for the panelists including additional information thereby allowing them to refine their statements from previous rounds (Procter & Hunt, 1994). This process produced 71 competency statements that comprised the pilot Peer Support Supervision Competency Survey.

### Pilot Peer Support Supervision Competency Survey

The pilot survey consisted of 15 demographic items and 71 competency statements that spanned the five-function model. The competency statements were organized as follows: Administrate (22 items), Educate (9 items), Support (18 items), Evaluate (8 items), and Advocate (14 items). The importance, criticality, and frequency of those competency statements were rated by survey respondents. Importance was evaluated by: *How important is the task to successfully perform the job?* (0 = Not Performed, 1 = Not Important, 2 = Somewhat Important, 3 = Important, 4 = Very Important). Frequency was evaluated by: *How often does a supervisor of peer support workers perform the task?* (0 = Never 1 = Every Few Months, 2 = Monthly, 3 = Weekly, 4 = Daily). Criticality was evaluated by: *What is the risk for an adverse consequence if the supervisor of peer support workers does not perform the task?* (0 = Competency not necessary, 1 = Very little risk or consequence, 2 = Moderate level of risk or consequence, 3 = High level of risk and consequence, 4 = Very high level of risk and consequences).

For item comparability, these three assessments were combined to create a composite impact weight. These impact weights were computed by adding the importance and criticality values and multiplying that sum by the frequency value [(I + C) × F] (Foglesong et al. 2022a, b; Sanchez & Fraser 1992; Kane et al., 1989). This analysis allowed calculation of a mean impact weight for each competency item. The mean impact weights for all items within a supervisory function (e.g., Administrate) were converted to Z scores for comparison of the items within each supervisory function.

#### Respondents

Of 330 survey responses, 100 were complete. Partially completed surveys were excluded from the analyses. 56% of the survey participants reported as PSS supervisors. 21% (*n* = 21) of participants were male, 65% (*n* = 65) female, and 14% reported non-binary/non-conforming, transgender, preferred not to answer or missing. 62% of participants (*n* = 62) were White or Caucasian, 13% (*n* = 13) Brown/African American, 8% (*n* = 8) Hispanic/Latinx/Spanish, and 15% (*n* = 15) reported mixed race, Hawaiian/other Pacific Islander, or preferred not to respond. 90% (*n* = 90) of survey respondents reported lived experience of mental illness, 8% (*n* = 8) reported no lived experience, and two participants preferred not to respond to this item.

#### Procedure

The pilot survey was developed in the online software Qualtrics and disseminated to PSS and PSS supervisors nationally via multiple listservs, websites, and direct email. The survey was distributed on the Academy of Peer Services (APS) listserv, the APS Virtual Community listserv, the Wellnessworks4us listserv, the Mental Health Consumers’ Self-Help Clearinghouse and National Coalition for Mental Health Recovery listserv. The survey was also sent by direct email to PSS and supervisors who were known to have an interest in further defining the competencies of supervision of the peer support workforce. The survey was open for a period of four weeks in June 2022.

## Results

Supervisory competencies varied in their perceived importance across the five functions of supervision (see Table 1). Clear patterns emerged in relation to relational, role-clarifying, and evaluative responsibilities. Across domains, the relationally grounded competencies consistently received the highest ratings. For example, within the Support function, items emphasizing core peer support values including active listening, confidentiality, and mutuality and transparency were rated among the most important supervisory behaviors overall. Similarly, within the Educate and Advocate functions, tasks such as modeling a non-judgmental stance and leading by example through empathy, curiosity, and a strengths focus ranked at the top of their respective categories, underscoring the value of relational presence and values-based leadership in supervision.

**Table 1:** Supervision Competencies

Description	Mean Weight	Z Score	*n*
Administrate			
Clarifies expectations of the PSS worker role	39.76	1.43	93
Explains the role and tasks of the PS worker in accordance with the job description	39.68	1.38	92
Provides training for new staff	39.60	1.34	95
Provides access to resource to maintain peer support skills	39.15	1.15	91
Orients new staff to the organization culture and mission	39.06	1.13	94
Interprets organizational policies and procedures for staff	34.18	(-0.98)	89
Conducts performance evaluations in accordance with intervals specified in the policies and procedures	34.16	(-0.99)	91
Advises on, or creates the job description, tasks and competencies	33.43	(-1.31)	93
Analyzes data based on deliverables for reporting and quality assurance	32.61	(-1.66)	92
Administers disciplinary actions in collaboration with HR	32.20	(-1.83)	90
Support			
Listens actively	43.34	1.28	89
Discusses the importance of confidentiality	43.33	1.25	91
Builds trust through mutuality and transparency	42.92	1.10	91
Encourages PSS to create learning opportunities for other staff	35.50	(-1.58)	94
Redirects staff seeking professional support to appropriate resources	35.31	(-1.65)	90
Utilizes institutionally conferred power to uplift staff	34.96	(-1.78)	89
Educate			
Models poise, listening, support and non judgmental stance	43.24	1.67	92
Discusses peer support history and practice	33.95	(-1.83)	91
*Advocate*			
Leads by example through empathy, curiosity and strengths focus	42.40	2.04	91
Ensures the PSS role retains its identity within the workplace	40.03	0.96	90
Acknowledges and navigates power differentials	34.97	(-1.34)	91
Provides opportunities for PSS role to develop and expand to a career ladder	34.57	(-1.52)	91
Evaluate			
Uses formal, informal and situational specific approaches to supervision	39.63	1.24	90
Implements a strengths-focused, standardized, and objective performance evaluation tool	34.37	(-0.99)	92
Creates, or suggests revisions to, the PSS job description to match values, principles, ethics & guidelines	33.11	(-1.54)	92

Administrative tasks reflected a variable pattern. Responsibilities related to role clarity and onboarding, such as explaining the peer support worker role, providing training for new staff, and orienting staff to organizational culture and mission, were rated as highly important. In contrast, more compliance-oriented activities including interpreting policies, conducting formal performance evaluations, and administering disciplinary actions were rated lower relative to other administrative tasks. Within the Evaluate function, supervisors placed greater importance on flexible and context-responsive approaches to supervision than on formalized evaluation tools alone. The use of formal, informal, and situational supervision strategies was rated higher than tasks related to standardized performance evaluation instruments or revisions to job descriptions, suggesting a preference for evaluative practices that are adaptive rather than exclusively procedural. Finally, advocacy-related tasks revealed a possible tension between values-driven leadership and PSS role advancement. The lowest rated competency in this domain was *Provides opportunities for PSS role to develop and expand to a career ladder*. While leading through empathy and ensuring the integrity of the peer support role were rated as highly important, activities focused on navigating power differentials and creating career ladder opportunities were rated lower.

## Discussion

These findings identify some potentially critical elements in the effective supervision of the peer support workforce. The 25 specific supervision competencies identified topics that warrant more or sometimes less emphasis in supervision as a first step towards guidelines for standardized peer supervisor training curricula and the creation of practice guidelines for supervisors. Conveying aspects of the peer supervisory task that should be emphasized, and other aspects that may warrant less emphasis, can make training more effective and efficient. As Sewell et al. (2024) demonstrate, when supervision is comprehensive and role-specific, organizations experience a significant increase in practitioner competence and professional well-being.

The utilization of the Five Critical Functions of Supervision model suggests that effective peer support supervision may require emphasizing elements such as advocacy that regular supervision may not. In the same vein, these results suggest that effective peer supervision may require emphasizing or deemphasizing topics differentially from traditional supervision. While impact scores reveal both high and low ranked competencies, both can inform training curricula for PSS supervisors. For this initial pilot survey, using approximately one standard deviation above or below the mean provided an easily understandable method for identifying topics that survey participants rated as more or less impactful. Clearly, competencies rated as impactful should be emphasized in peer supervision training. Conversely, competencies rated as less impactful might be deemphasized, but their meaning may be less clear. These competencies were initially identified through a Delphi process before being rated for their impact, hence positive and negative impact ratings are treated as equally salient. Some considerations for lower impact scores are that the respondent may have found the competency less within their control (i.e. Writing a job description), less important to their role (i.e. Analyzes data based on deliverables for reporting and quality assurance), or less critical meaning it would not cause harm if not prioritized (i.e. Encourages peer support staff to create new learning opportunities for other staff). A competency may have also been ranked low because the supervisor might not have the requisite knowledge or experience to perform the task (i.e. Acknowledges and navigates power differentials). Whatever the reason for the lower ranking, the competency is still a valuable metric for understanding the full scope of supervision. Focusing solely on the most highly ranked competencies would limit the understanding and scope of the supervision process.

### Limitations

Limitations of the focus group protocol and design were noted. Panelists were more familiar with the administrative, educative, and supportive functions of supervision; whereas tasks ascribed to the advocative and evaluative functions were described but not previously categorized using those specific terms. Responses indicated that specific supervision competencies were associated with more than one function. This finding influenced how later described functions (e.g., advocative) may be perceived with respect to earlier discussed functions (e.g., administrative). PSS were involved later in the process when asked to review the competencies generated by the PSS supervisors. We surmise that the PSS may have identified different competencies had they been involved earlier. We found a lack of uniformity among job titles across service settings, agencies, regions, and states. This could be attributed to survey design and item clarity. The length of the survey was likely too burdensome to complete, resulting in survey fatigue as evidenced by 100 of the 330 pilot surveys completed. Finally, we noted that some of the competency statements included terminology that was esoteric. This likely resulted in skipped items and items rated based on inaccurate comprehension.

## Conclusion

By utilizing a Participatory Action Research (PAR) approach, the study tapped the experiential wisdom of both PSS supervisors and PSS to define what effective supervision looks like in practice. The results demonstrate supervisory tasks that highlight role clarity, active listening, and leading through empathy, while also identifying a significant need for training in the domains of advocacy and evaluation. These findings suggest that approaches to supervision of peer support specialists can be distinguished from approaches utilized with clinical staff to recognize and account for unique concerns of the peer support specialist role. The supervision competencies identified in this study may provide an initial step in creating a blueprint for developing training curricula and competencies for PSS supervisors. Future research may focus on testing the validity of the Five Critical Functions of Supervision model. Effective peer supervision is essential for increasing job satisfaction and retention, ensuring that peer support remains a viable, integral and sustainable discipline within the healthcare landscape. 

## Data Availability

No datasets were generated or analysed during the current study.

## References

[CR1] Abraham, K., Erickson, P., Sata, M., & Lewis, S. (2021). Job satisfaction and burnout among peer support specialists: The contributions of supervisory mentorship, recovery-oriented workplaces, and role clarity. *Advances in Mental Health Promotion, Prevention, and Early Intervention*, *20*(1), 38–50. 10.1080/18387357.2021.1977667

[CR2] Alberta, A., Ploski, R., & Carlson, S. (2012). Addressing challenges to providing peer-based recovery support. *Journal of Behavioral Health Services & Research*, *39*(4), 481–491. 10.1007/s11414-012-9286-y 22828975

[CR3] Battel-Kirk, B., Barry, M. M., Taub, A., & Lysoby, L. (2009). A review of the international literature on health promotion competencies: Identifying frameworks and core competencies. *Global Health Promotion*, *16*(2), 12–20. 10.1177/175797590910410 19477859

[CR4] Bennetts, W., Pinches, A., Paluch, T., & Fossey, E. (2013). Real lives, real jobs: Sustaining consumer perspective work in the mental health sector. *Advances in Mental Health*, *11*(3), 313–326.

[CR5] Cabral, L., Strother, H., Muhr, K., Sefton, L., & Savageau, J. (2014). Clarifying the role of the mental health peer specialist in Massachusetts, USA: Insights from peer specialists, supervisors and clients. *Health Social Care Community*, *22*(1), 104–112. 10.1111/hsc.12072 24313729

[CR6] Centers for Medicare & Medicaid Services (2007). cms.gov/cmsgov/archived-downloads/smdl/downloads/smd081507a.pdf 26110197

[CR7] Centers for Medicare & Medicaid Services (2024). Frequently Asked Questions on Medicaid and CHIP Coverage of Peer Support Services, https://www.medicaid.gov/federal-policy-guidance/downloads/faq06052024.pdf

[CR8] Chinman, M., & Al. (2014a). Peer support services for individuals with serious mental illnesses. *Assessing the evidence Psychiatric Services*, *65*(4), 429–441. 10.1176/appi.ps.201300244 24549400

[CR9] Chinman, M., Henze, K., & Sweeney, P. (2014b). Peer specialist toolkit: Implementing peer support services in VHA. VISN 1 New England MIRECC Peer Education Center and VISN 4 MIRECC Peer Resource Center. VA Health Services Research & Development. *Dept of Veterans Affairs*. https://www.mirecc.va.gov/visn4/docs/peer_specialist_toolkit_final.pdf

[CR10] Cronise, R., Teixeira, C., Rogers, E. S., & Harrington, S. (2016). The peer support workforce: Results of a national survey. *Psychiatric Rehabilitation Journal*, *39*(3), 211–221. 10.1037/prj0000222 27618458

[CR11] Crutzen, R., de Nooijer, J., Brouwer, W., Oenema, A., Brug, J., & de Vries, N. K. (2008). Internet-delivered interventions aimed at adolescents: A Delphi study on dissemination and exposure. *Health Education Research*, *23*(3), 427–439. 10.1093/her/cym094 18209115

[CR12] Daniels, A. S., Tunner, T. P., Powell, I., Fricks, & Ashenden, P. (2015). Pillars of peer support-VI: Peer specialist supervision. https://copelandcenter.com/pillars-peer-support

[CR13] Davis, J. (2013). Predictors of job satisfaction among peer providers on professional treatment teams in community-based agencies. *Psychiatric Services*, *64*(2), 181–184. 23370625 10.1176/appi.ps.001452012

[CR14] Davis, J. (2015). Supervision of peer specialists in community mental health center: Practices that predict role clarity. *Social Work in Mental Health*, *13*(2), 145–158. 10.1080/15332985.2013.801390

[CR15] Delman, J., & Klodnick, V. (2016). Factors supporting the employment of young adult peer providers: Perspectives of peers and supervisors. *Community Mental Health Journal*, 1–12. 10.1007/s10597-016-0059-6 27770306

[CR16] Edwards, J. P. (2023, December 4). Do I Know You? Do You Know Me? Bridge Building Through Shared Learning [Keynote Speech]. Legere Des Heils National Inaugural Peer Support Day Conference, Lunteren, Netherlands.

[CR17] Edwards, J. P. (2024, March 15). Five Critical Functions of Supervision: Competencies of Effective Supervision [Keynote Presentation]. Supervision Summit, Peer Support Technical Assistance Center (PeerTAC), Virtual Conference.

[CR18] Edwards, J. P. (2025)*.* Supervising, Retaining, and Advancing the Careers of Lived Experience Workers [Invited Presentation]. The Living and Lived Experience Workforce Learning Collaborative (LLEWLC) in partnership with Yale Program. for Recovery and Community Health (PRCH), Virtual Forum.

[CR19] Edwards, J. P., & Foglesong, D. (2024, April 15). Supervising Peer Specialists in Changing Times [Workshop Presentation]. NATCON24, National Council for Mental Wellbeing, St. Louis, MO, United States.

[CR20] Edwards, J. P., & Ikuta, Y. (2016). Supervision Strategies: A Dialogue on Best Practices for Supervising Peer Supporters [Conference Workshop]. International Association of Peer Supporters (INAPS) Conference, Philadelphia, PA. August

[CR21] Edwards, J. P., & Solomon, P. (2023). Explaining job satisfaction among mental health peer support workers. *Psychiatric Rehabilitation Journal*, *46*(3), 223–231. 10.1037/prj0000577 37470983

[CR22] Edwards, J. P., Enders, G., & Forbes, J. (2022)*. *National Practice Guidelines for Peer Specialists and Supervisors and The Five Critical Functions of Supervision. [Presentation]. ECHO Series, Dartmouth University.

[CR23] Edwards, J. P., Spagnolo, A., Cronise, R., Enders, G., Forbes, J., & Pratt, C. (2024, January 31). The Future of Peer Support Supervision: Findings from Participatory Action Research [Conference Presentation] SHARE! Self Help and Society for Community Research and Action Conference on Supervision, Virtual Presentation.

[CR24] Foglesong, D., Spagnolo, A., Cronise, R., Forbes, J., Swarbrick, P., Edwards, J., & Pratt, C. (2022a). Perceptions of supervisors of peer support workers (PSW) in behavioral health: Results from a national survey. *Community Mental Health Journal*, *58*(3), 437–443. 10.1007/s10597-021-00837-2 34089113 PMC8177034

[CR25] Foglesong, D., Knowles, K., Cronise, R., Wolf, J., & Edwards, J. P. (2022b). National Practice Guidelines for Peer Support Specialists and Supervisors. *Psychiatric Services*, *73*(2), 215–218. 10.1176/appi.ps.202000901 34253034

[CR26] Forbes, J., Pratt, C., & Cronise, R. (2022). Experiences of peer support specialists supervised by nonpeer supervisors. *Psychiatric Rehabilitation Journal*, *45*(1), 54–60. 33793288 10.1037/prj0000475

[CR27] Green, L., & Kreuter, M. (1999). *Health promotion planning: An educational and environmental approach* (2nd ed.). Mayfield Publishing Co.

[CR28] Hasson, F., Keeney, S., & McKenna, H. (2000) Research guidelines for the e-Delphi survey technique. *Journal of Advanced Nursing, 32*(4),1008–1015. 11095242

[CR29] Hino, S. (2014). A unique but ambiguous role. *Peers for Progress*. http://peersforprogress.org/pfp_blog/what-is-a-mental-health-peer-specialists-role-in-a-care-team/

[CR30] Iqbal, S., & Pipon Young, L. (2009). The e-Delphi method. *The Psychologist*, *22*, 599–601.

[CR31] Janke, K. K., Kelley, K. A., Sweet, B. V., & Kuba, S. E. (2016) A modified Delphi process to define competencies for assessment leads supporting a doctor of pharmacy program. *American Journal of Pharmaceutical Education*, *80*(10), Article 167. 10.5688/ajpe8010167 PMC5289723 28179716

[CR32] Kane, M., Kingshury, C., Colton, D., & Estes, C. (1989). Combining data on criticality and frequency in developing test plans for licensure and certification exams. *Journal of Educational Measurement*, *26*(1), 17–27. 10.1111/j.1745-3984.1989.tb00315.x

[CR33] Karyczak, S., Spagnolo, A., Higbee, S., et al. (2025). Identifying the Core Competencies for Crisis Peer Support Specialists: an e-Delphi Study. *Community Mental Health*. 10.1007/s10597-025-01573-7 41417140

[CR34] Mancini, M. (2019). Strategic storytelling: An exploration of the professional practices of mental health peer providers. *Qualitative Health Research*, *29*(9), 1266–1276. 10.1177/1049732318821689 30616464

[CR35] Mead, S., & McNeil, C. (2004). Peer support: What makes it unique? *The International Journal of Psychosocial Rehabilitation*, *10*(2), 29–37.

[CR36] Mead, S., Hilton, D., & Curtis, L. (2001). Peer support: A theoretical perspective. *Psychiatric Rehabilitation Journal*, *25*(2), 134–141. 11769979 10.1037/h0095032

[CR37] Mental Health America (2018). Evidence for peer support. *Mental Health America Summary of Research* https://www.mhanational.org/sites/default/files/Evidence%20for%20Peer%20Support%20May%202018.pdf

[CR38] Miyamoto, Y., & Sono, T. (2012). Lessons from peer support among individuals with mental health difficulties: A review of the literature. *Clinical Practice Epidemiology Mental Health*, *8*, 22–29. 10.2174/1745017901208010022 PMC3343315 22563347

[CR39] Moran, G. S., Russinova, Z., Gidugu, V., et al. (2013). Challenges experienced by paid peer providers in mental health recovery: A qualitative study. *Community Mental Health Journal*, *49*, 281–291. 10.1007/s10597-012-9541-y 23117937

[CR40] Mowbray, C. T., Moxley, D. P., Thrasher, S., et al. (1996). Consumers as community support providers issues created by role innovation. *Community Mental Health Journal*, *32*(1), 47–67. 8635317 10.1007/BF02249367

[CR41] Myrick, K., & del Vecchio, P. (2016). Peer support services in the behavioral healthcare workforce: State of the field. *Psychiatric Rehabilitation Journal*, *39*(3), 197–203. 10.1037/prj0000188 27183186

[CR42] N.A.P.S. (2013). National practice guidelines for peer supporters. *International Association of Peer Supporters. (N.A.P.S.)* Ada, Michigan.https://www.peersupportworks.org/wpcontent/uploads/2021/02/nationalguidelines_updated.pdf

[CR43] N.A.P.S. (2019). National practice guidelines for peer specialists and supervisors. *National Association of Peer Supporters (N.A.P.S.)*, Washington, DC.https://www.peersupportworks.org/wp-content/uploads/2021/07/National-Practice-Guidelines-for-Peer-Specialists-and-Supervisors-1.pdf

[CR44] NMAC National Minority AIDS Council (2015). From start to finish: An interactive guide to focus groups. Retrieved from: https://thecarecouncil.org/wp-content/uploads/2015/01/From-Start-to- Finish-Focus-Group-Workbook.pdf.

[CR45] Nyumba, T. O., Wilson, K., Derrick, C. J., & Mukherjee, N. (2018). The use of focus group discussion methodology: Insights from two decades of application in conservation. *Methods in Ecology and Evolution*, *9*(1). 10.1111/2041-210X.12860

[CR46] Onwuegbuzie, A., Dickinson, W. B., & Leech, N. L. (2009). A qualitative framework for collecting and analyzing data in focus group research International. *Journal of Qualitative Methods*, *8*(3), 1–21 10.1177/160940690900800301

[CR47] Peer Recovery Center of Excellence (2023). Comparative analysis of state requirementsfor peer support specialist training and certification in the United States. https://peerrecoverynow.org/documents/Final.Comparative.Analysis.pdf

[CR48] Procter, S., & Hunt, M. (1994). Using the e-Delphi survey technique to develop a professional definition of nursing for analyzing nursing workload. *Journal of Advanced Nursing*, *19*, 1003–1014. 8056906 10.1111/j.1365-2648.1994.tb01180.x

[CR49] Reuling, J., Cronise, R., & Wolf, J. (2024). Listening to the Peer Support Workforce – Top Ten Priorities: An Action Agenda. https://tucollaborative.org/peersupport/listening-to-the-peer-support-workforce/

[CR50] Salzer, M., Schwenk, E., & Brusilovsky, E. (2010). Certified peer specialists roles and activities: Results from a national survey. *Psychiatric Services, 61(*5), 520–523. 10.1176/ps.2010.61.5.520 20439376

[CR51] SAMHSA. (2015). BRSS TAC. Core competencies for workers in behavioral health settings. https://www.samhsa.gov/sites/default/files/programs_campaigns/brss_tacs/core-competencies_508_12_13_18.pdf

[CR52] SAMHSA. (2017). Value of peers infographic: Peer supporting recovery from mental health conditions. SAMHSA Bringing Recovery Supports to Scale Technical Assistance Center Strategy(BRSS TACS). https://www.samhsa.gov/sites/default/files/programs_campaigns/brss_tacs/peers-supporting-recovery-mental-health-conditions-2017.pdf

[CR53] SAMHSA. (2020). Behavioral health workforce report. Substance Abuse and Mental Health Services Administration available through the Annapolis Coalition. https://annapoliscoalition.org/samhsa-releases-behavioral-health-workforce-report/

[CR54] SAMHSA. (2023). National Model Standards for Peer Support Certification. https://web.archive.org/web/20240805175329/https://store.samhsa.gov/sites/default/files/pep23-10-01-001.pdf

[CR55] SAMHSA, B. R. S. S. T. A. C. S. (2018). Guidelines for peer supervision. https://www.samhsa.gov/sites/default/files/programs_campaigns/brss_tacs/guidelines-peer-Supervision-4-ppt-cp5.pdf

[CR56] SAMHSA, BRSS TACS (n.d). Supervision of Peer Workers. https://www.samhsa.gov/sites/default/files/programs_campaigns/brss_tacs/guidelines-peer-supervision-4-ppt-cp5.pdf

[CR57] Sanchez, J. I., & Frazer, S. L. (1992). On the choice of scales for task analysis. *Journal of Applied Psychology*, *77*, 545–553.

[CR58] Sewell, K. M., McMenemy, C., van Rensburg, M. J., & MacDonald, H. (2024). Organizational outcomes of supervision within human services: A scoping review. *Human service organizations: Management leadership & governance*, *48*(1), 19–42.

[CR59] Slocum, N. (2005). Participatory Methods Toolkit. A practitioner’s manual. King Baudouin Foundation and the Flemish Institute for Science and Technology Assessment. ISBN 90-5130-506-0.

[CR60] Smith, D. (2007, August 15). Letter to state Medicaid directors to provide guidance on peer support services under the Medicaid program. Centers for Medicare and Medicaid Services (CMS), Baltimore, MD. https://downloads.cms.gov/cmsgov/archived-downloads/smdl/downloads/smd081507a.pdf

[CR62] Stephancic, A., Bochicchio, L., Tuda, D., Harris, Y., DeSomma, K., & Cabassa, L. (2021). Strategies and lessons learned for supporting and supervising peer specialists. *Psychiatric Services*, *72*(5). 10.1176/appi.ps.202000515 PMC8656242 33657843

[CR63] Strauss, A., & Corbin, J. (1998). Basics of Qualitative Research: Technique and Procedures for Developing Grounded Theory. Sage Publications. Thousand Oaks, CA. ISBN 0-8039-5939-7.

[CR64] University of Illinois, College of Medicine: Center on Mental Health Services Research and Policy (2025). National Survey on the Health Status of the Certified Peer Specialist (CPS) Workforce. https://www.psych.uic.edu/research/center-on-mental-health-services-research-and-policy/our-research/national-survey-on-the-health-status-of-the-certified-peer-specialist-cps-workforce#page-start

[CR65] White, S., Foster, R., Marks, J., Morshead, R., Goldsmith, L., Barlow, S., Sin, J., & Gillard, S. (2020) The effectiveness of one-to-one peer support in mental health services: A systematic review and meta-analysis. *BMC Psychiatry*, 20, 534. 10.1186/s12888-020-02923-3 PMC7657356 33176729

[CR66] Wolf, J. (2018). National trends in peer specialist certification. *Psychiatric Services*, *69*(10), 1049. 10.1176/appi.ps.201800333 30277436

